# Possible Usefulness of Gadolinium-Enhanced Brain MRI for Evaluating Risk of Perioperative Hemorrhage: A Case of Infective Endocarditis

**DOI:** 10.1155/2014/158041

**Published:** 2014-02-13

**Authors:** Chikahiko Koeda, Atsushi Tashiro, Tomohiro Takahashi, Masanobu Niiyama, Ryohei Sakamoto, Takumi Kimura, Yoshihiro Morino, Katsutoshi Terui, Ryoichi Tanaka, Kunihiro Yoshioka, Hajime Kin, Hitoshi Okabayashi, Motoyuki Nakamura

**Affiliations:** ^1^Division of Cardioangiology, Department of Internal Medicine, Iwate Medical University, 19-1 Uchimaru, Morioka 020-8505, Japan; ^2^Department of Emergency, Iwate Medical University, 19-1 Uchimaru, Morioka 020-8505, Japan; ^3^Department of Radiology, Iwate Medical University, 19-1 Uchimaru, Morioka 020-8505, Japan; ^4^Department of Cardiovascular Surgery, Iwate Medical University, 19-1 Uchimaru, Morioka 020-8505, Japan

## Abstract

A 59-year-old woman visited a local hospital for fever and was diagnosed as having infective endocarditis (IE) on the basis of blood cultures and transthoracic echocardiography. Based on clinical episodes of subarachnoid hemorrhage after admission, it was judged that she was not a good candidate for urgent open heart surgery, and it was decided to treat her with conservative medical therapy for the acute phase. We explored the optimum timing for surgery by employing gadolinium (Gd) contrast medium-enhanced magnetic resonance imaging (MRI) T2* weighted image (black dots) due to her high risk of perioperative cerebral hemorrhage. After the disappearance of the contrast media enhancement effect around the black dots, open heart surgery was performed successfully on the 103rd hospitalization day. The patient was discharged 22 days after the surgery with no clinical complications. This case suggests that disappearance of the contrast media enhancement effect around the black dots may be a useful marker for optimal timing of surgery to minimize the risk of perioperative cerebral hemorrhage in patients with IE. *Learning Objective*. The MRI T2* weighted images including those with Gd contrast medium enhancement effect may be useful for evaluating the risk of perioperative intracranial hemorrhage in IE.

## 1. Introduction

Infective endocarditis (IE) is a serious disease characterized by refractory heart failure combined with sepsis. Intracranial bleeding is an important potential complication which can be influenced by the timing of open heart surgery [1]. Here we report a case of IE complicated with a brain mycotic aneurysm that was checked regularly by brain magnetic resonance imaging (MRI) with gadolinium (Gd) enhancement to explore optimal timing for open chest surgery. As a result, after disappearance of Gd-enhanced T2* weighted image (black dots), the patient underwent successful valve replacement without perioperative symptomatic complications. We discuss the possible usefulness of Gd-enhanced brain MRI to minimize perioperative hemorrhagic complications based on existing knowledge in the literature.

## 2. Case Report

A 59-year-old woman had an intermittent fever from the end of October to the end of November 2012 when she consulted a local hospital and *Streptococcus viridans *was detected by blood culture. Transthoracic echocardiography (TTE) revealed the presence of vegetations on the aortic and mitral valves with regurgitation. On the basis of the Duke criteria, she was diagnosed with definite infective endocarditis (IE), and intravenous antibiotic treatment (penicillin G 18 × 10^6^ units/day + gentamicin 80 mg/day) was started. She was admitted to our hospital on December 6, 2012.

On presentation, her clinical characteristics were height 155 cm, weight 50.9 kg, blood pressure 141/76 mmHg, heart rate 93 beats per minute regular, body temperature 36.4°C, SpO_2_ (room air) 96%, and heart sound systolic murmur (Levine IV). Blood test data were as follows: WBC 6.17 × 10^3^/*μ*L, C-reactive protein 1.7 mg/dL, aspartyl aminotransferase 29 IU/L, alanine aminotransferase 17 IU/L, lactate dehydrogenase 276 IU/L, blood urea nitrogen 3.9 mg/dL, and serum creatinine (CRE) 1.18 mg/dL. Chest X-ray showed a cardiothoracic ratio of 60% with lung congestion and pleural fluid. TTE and transesophageal echocardiography revealed a left ventricular dimension (diastole/systole) of 5.0/2.7 cm, ejection fraction of 73%, and an estimated systolic right ventricular pressure of 75 mmHg. The vegetation on the anterior mitral leaflet and noncoronary cusp of the aortic valves was found with regurgitation due to ruptured chordae tendineae. The vegetation size was 8–10 mm.

Brain MRI T2* weighted images revealed black dots on the right frontal lobe, both sides of the parietal lobe and the right occipital lobe, and a localized subarachnoid hemorrhage in the right postcentral gyrus. An enhancement effect with gadolinium (Gd) contrast media was seen in the dot on the right occipital lobe. According to recent reports, this type of MRI imaging may indicate fragility of the arterial wall of a brain mycotic aneurysm [2, 3]. As a consequence, it was judged that the patient was not a good candidate for urgent open heart surgery because of a potentially high risk for perioperative subarachnoid hemorrhage.

**Figure 1:**
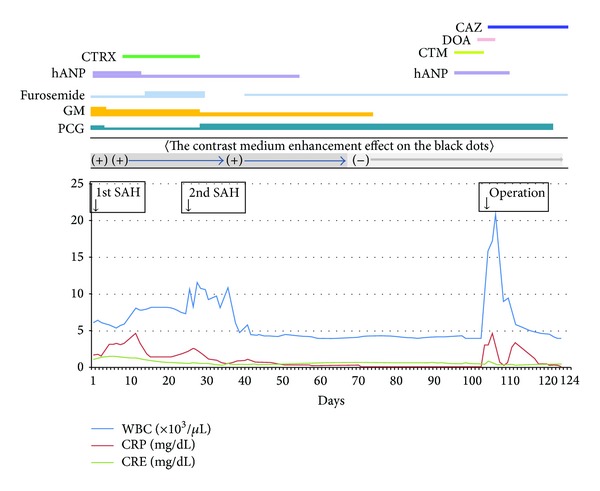
Clinical course. hANP = human atrial natriuretic peptide, GM = gentamicin, PCG = penicillin, SAH = subarachnoid hemorrhage, CTRX = ceftriaxone, CAZ = ceftazidime, DOA = Dopamine, and CTM = cefotiam.

**Figure 2:**
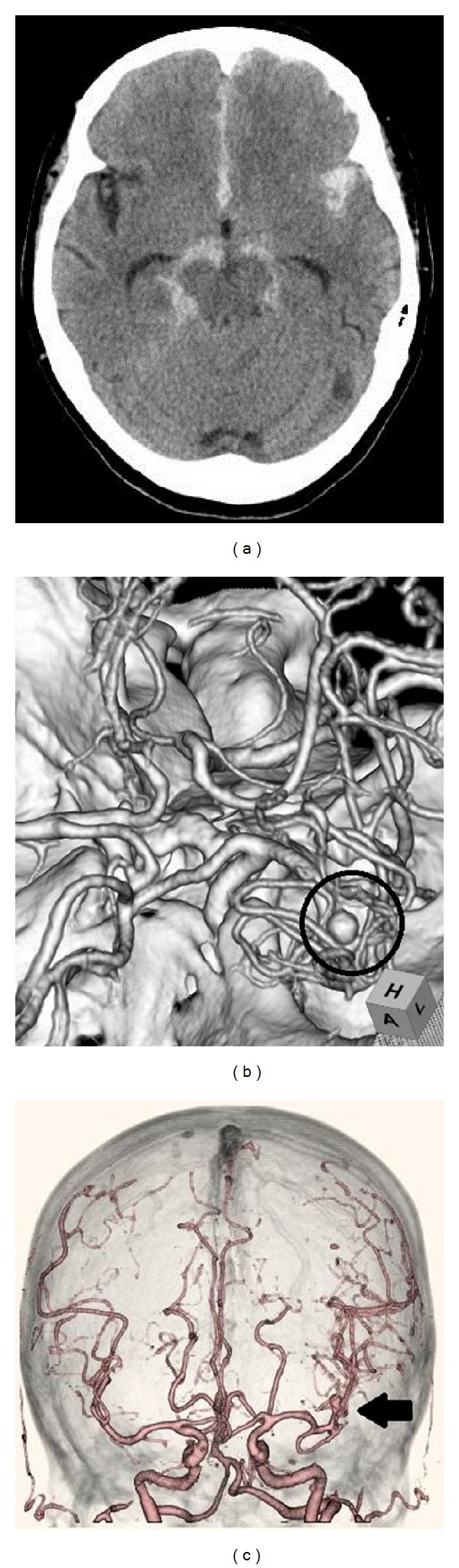
A subarachnoid hemorrhage (a) originating from the cerebral aneurysm on the left middle cerebral artery (b, c).

**Figure 3:**
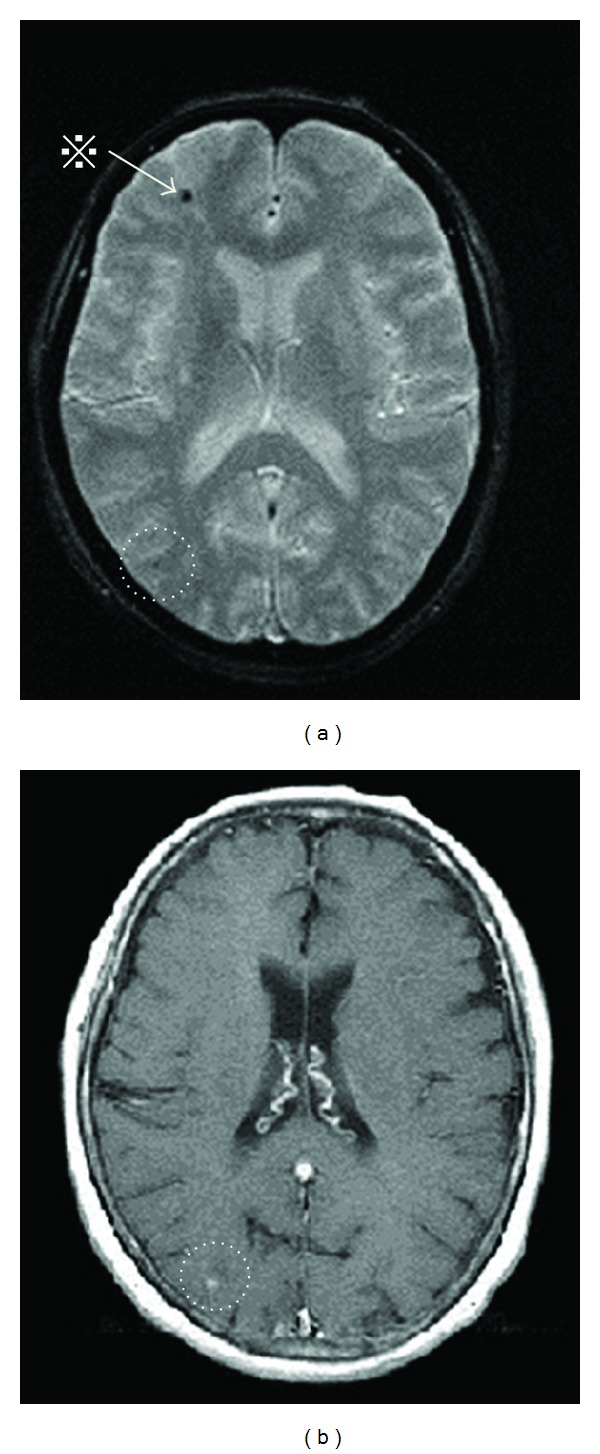
Black dots on T2* weighted brain magnetic resonance imaging (a) and the gadolinium (Gd) contrast medium enhancement effect. (b) *※* a black dot on the right frontal lobe does not show the enhancement effect seen at (b) (i.e., no fresh microbleeding).

Therefore, conservative medical treatment was started with carperitide, furosemide, and antibiotic for the acute phase. The acute phase passed without worsening of the heart failure, but subarachnoid hemorrhage developed again on the 26th day after admission (Figure 1). A 4 mm saccular cerebral mycotic aneurysm was observed in the left middle cerebral artery on computed tomography (CT) angiography (Figure 2). We considered a choice of endovascular embolization for the mycotic aneurysm; however, there was a concern that mechanical stimulation may result in an iatrogenic rupture of the aneurysm due to weakening of the vascular wall caused by active inflammation. Consequently, we waited for the aneurysm to become stable after being thrombosed by conservative medical treatment. We continued conservative medical treatment until the Gd contrast enhancement around the black dots disappeared (Figure 3). After the enhancement effect had disappeared, the patient underwent successful elective open heart surgery on the 103rd day of hospitalization with no surgical complications. After surgery, she had a minor asymptomatic complication of a small subdural hematoma on the left parietal lobe, but there were no neurological deficit symptoms, and she was discharged 22 days after surgery.

### 2.1. Surgical Findings

Small valvular vegetations were detected on the leaflet edges of the aortic tricuspid valves and were resected. A vegetation was detected on the ruptured chordae tendineae of the middle scallop of the posterior leaflet (P2) of the mitral valve. In addition, we resected another vegetation and thickening of the posterior leaflet (P3) and the anterior leaflet. The procedure was completed by mitral annuloplasty only because valve function was preserved. The cardiopulmonary bypass and aortic cross-clamp time was 134 minutes.

### 2.2. Pathological Findings

The mitral valve posterior leaflet was infiltrated by lymphocytes and consisted of irregular fibrotic tissues. Inflammatory fibrous connective tissue with calcification was observed in the aortic valve left coronary cusp. Granular tissue with calcification and multinucleated giant cells were observed in the noncoronary aortic valve cusp.

## 3. Discussion

The outcome of IE is occasionally poor due to septic complications and refractory heart failure resulting from valvular destruction. Several reports have demonstrated that early open heart surgery may be desirable in patients with IE [4]. However, there are problems of secondary infection and hemorrhagic complications in the perioperative period, and there is no consensus regarding the optimal timing of open heart surgery [5]. The complication of intracranial bleeding is the most important concern related to cardiopulmonary bypass surgery, and this is a critical factor leading to postponement of radical surgery [1]. However, useful markers for accessing the risk of intracranial bleeding during and after surgery for IE have yet to be established.

The “black dot” (otherwise called “Bull's eye-like lesion”) on the MRI T2* weighted image suggests the presence of hemosiderin due to microbleeding from a cerebral artery [6–8]. The finding of “black dot” occurs in a high proportion of IE cases and is associated with mycotic aneurysm rupture [2, 3]. It is thought that the pathological condition in the cerebral arterial wall that results in the black dot is similar to the pseudoaneurysm proposed by Bohmfalk and others [9]. This mycotic cerebral aneurysm results from microbleeding and microembolus caused by septic conditions. Furthermore, when the enhancement effect of Gd contrast media appears on the black dots on MRI, this suggests that the vascular wall has become fragile [10]. It is therefore assumed that use of anticoagulants such as heparin during open heart surgery may generate a high risk for hemorrhagic complications. It is thus reasonable to assume that the risk of perioperative intracranial hemorrhage may have diminished after the disappearance of the contrast medium enhancement effect on the black dots [11]. On the basis of the present case report, it may be suggested that this clinical strategy (employing Gd-enhanced brain MRI imaging) may be valuable for optimizing the timing of cardiac surgery.

## 4. Conclusion

The MRI T2* weighted images including those with Gd contrast medium enhancement effect may be useful for evaluating the risk of perioperative intracranial hemorrhage in IE. On the basis of the present case, the disappearance of the contrast medium enhancement effect around the black dots may be a useful marker for optimizing the timing of surgery so as to minimize the risk of perioperative cerebral hemorrhagic complication in patients with IE.
